# Genome-Wide Identification of the *Rehmannia glutinosa* miRNA Family and Exploration of Their Expression Characteristics Caused by the Replant Disease Formation-Related Principal Factor

**DOI:** 10.3390/genes15091239

**Published:** 2024-09-23

**Authors:** Li Gu, Yanlin Lai, Guojun Zhang, Yanhui Yang, Bao Zhang, Jianming Wang, Zhongyi Zhang, Mingjie Li

**Affiliations:** 1 College of Bee Science and Biomedicine, Fujian Agriculture and Forestry University, Fuzhou 350002, China; 2 Key Laboratory of Ministry of Education for Genetics, Breeding and Multiple Utilization of Crops, Fujian Agriculture and Forestry University, Fuzhou 350002, China; 3 College of Bioengineering, Henan University of Technology, Zhengzhou 450001, China; 4 School of Pharmacy, Henan University of Chinese Medicine, Zhengzhou 450046, China

**Keywords:** *Rehmannia glutinosa*, miRNAs, replant disease, principal factors, FA, FO, *RgmiR398*

## Abstract

**Background/Objectives:** *Rehmannia glutinosa*, a highly valuable medicinal plant in China, is encountering severe replant disease. Replant disease represents a complex stress driven by multiple principal factors (RDFs), including allelochemicals, microbes, and their interactions. miRNAs are recognized as key regulators of plant response to stresses; however, their specific roles within RDFs are not entirely clear. **Methods:** This study builds six RDF treatments, comprising *R. glutinosa* continuously planted (SP), normally planted (NP), and NP treated with ferulic acid (FA), *Fusarium oxysporum* (FO), and a combination of FA with FO (FAFO). sRNA-seq technology was used to identify crucial miRNAs in response to diverse RDFs. **Results:** In total, 30 sRNA datasets were generated from the SP, NP, FA, FO, and FAFO samples. A total of 160 known and 41 novel miRNAs (*RgmiRNAs*) were identified in the *R. glutinosa* genome based on the sRNA database. Abundance analysis revealed that *RgmiRNAs* in SP exhibited a distinct expression profile in comparison with others. Of these, 124, 86, 86, and 90 *RgmiRNAs* were differentially expressed in SP, FA, FO, and FAFO compared with NP. Target analysis indicated that *RgmiRNAs* downregulated in both SP and RDFs impede the organism growth of *R. glutinosa*. *RgmiRNAs* upregulated in SP can disrupt root formation and nutrient metabolism, in which, two *RgmiR398* were uniquely expressed in SP. It was confirmed to target *RgCSD* genes. The expression patterns of *RgmiR398* and *RgCSD* indicated that replant disease induces the oxidative damage of *R. glutinosa* through *RgmiR398*. **Conclusions:** *RgmiRNA* profiling under RDFs provides a theoretical basis for the further clarification of *RgmiRNA* function in replant disease.

## 1. Introduction

*Rehmannia glutinosa* (Gaert.) Libosch. (*R. glutinosa*) is a perennial herb belonging to the *Rehmannia* genus of the Scrophulariaceae family [1], which comprises six species. Of these, only *R. glutinosa* is widely cultivated in China, with its tuberous roots employed as raw materials for traditional Chinese herbal medicine, *Rehmanniae radix* in Chinese Pharmacopoeia [1,2]. At present, a number of significant active ingredients have been identified in the tuberous roots, fibrous roots, and leaves of *R. glutinosa*, including monosaccharides, polysaccharides, phenylethanoid, iridoid glycosides, catalpol, and trichitosans. The in vitro and in vivo experiments have demonstrated that these ingredients exhibit a range of pharmacological activities, such as antitumour, immunomodulatory, cardiovascular, cardioprotective, cerebrovascular, and antioxidant effects [1,2,3]. In recent years, with the increase in market demand for *R. glutinosa* and the reduction in cultivated areas, *R. glutinosa* has begun to be monoculturally cultivated in a large area in production. However, the continuous planting of *R. glutinosa* in the same field will inevitably give rise to the emergence of consecutive monoculture problems (CMP), also referred to as replant disease (RD) [4,5]. The practice of replanting has resulted in a range of adverse effects on the growth and development of *R. glutinosa*, including stunted growth, the arrested formation of tuberous roots, an increased prevalence of plant diseases and insect pests, and higher death rates [4,5,6]. Currently, no effective method or technology is available to control replant disease.

The formation of replant disease cannot be attributed to a single factor; rather, it results from the interaction of multiple factors within the “plant-soil-microbe” system [7,8]. An increasing number of studies indicate that the continuous accumulation of autotoxic allelochemicals in the rhizosphere of plants is a key factor in the onset of replant disease [9,10,11]. The proliferation of rhizosphere pathogenic microbes has been observed to be promoted by the presence of autotoxic allelochemicals in *R. glutinosa* [12]. Further studies have demonstrated that the representative autotoxic allelochemical, ferulic acid, either when present alone or in combination with *R. glutinosa*-specific microbes, *Fusarium oxysporum*, has the potential to exert a detrimental effect on the growth of *R. glutinosa*. It is currently hypothesized that autotoxic allelochemicals, harmful microbes, and their interactions within the rhizosphere are the principal factors influencing replant disease formation (RDFs). Despite the identification of numerous RDFs in plants, the precise mechanisms by which they cause damage to replanted plants, particularly at the molecular level, remain unclear.

The early growth stage of *R. glutinosa* has been identified as a critical period for the development of replant disease, as evidenced by field studies [4]. At this stage, multiple omics has revealed that several key cellular processes, including reactive oxygen species (ROS) generation, ethylene signalling, calcium signalling, and chromatin modification, exhibit abnormal alterations in replant *R. glutinosa* [13,14,15]. Furthermore, a significant number of non-coding RNAs, particularly microRNAs, have been demonstrated to regulate these cellular processes [15]. Furthermore, miRNAs have been identified as key regulators of essential cellular processes in plants exhibiting symptoms of replant disease, as evidenced by studies on *Pogostemon cablin* [16], *Polygonatum odoratum* [17], and *Salvia miltiorrhiza* [18]. Consequently, recent advances have demonstrated that miRNAs facilitate the establishment of replant disease by regulating specific response genes. Replant disease is characterized by the presence of multiple factors, which form a complex stress environment in rhizospheres [8,9,10]. The evidence indicates that miRNAs play a crucial role in a wide range of biotic and abiotic stressors [19]. Accordingly, it can be postulated that the number of miRNAs should be greater in replant disease than in single stress factors. In a previous study, a preliminary screening of a list of *R. glutinosa* miRNAs related to the formation of replant disease was conducted in the field [15]. The precise role of these miRNAs in allelochemicals, autotoxins, specific microbes, and their interactions remains ambiguous, and their specific role in the formation of replant disease cannot be ascertained. It is therefore of the utmost importance to analyze the expression variances of miRNA in single factors, multi-factors, and compound factors, as well as in real replant soils, with the aim of identifying the specific miRNA that responds to actual replant disease.

A fundamental prerequisite for the study of miRNAs lies in the comprehensive identification of the mature and precursor sequence of miRNA families. Previous studies have encountered considerable challenges in identifying *R. glutinosa* miRNA precursors due to the lack of genomic data available for *R. glutinosa*. In 2021, Ma et al. [20] published the genome sequence of *R. glutinosa*, thereby providing substantial support for the more comprehensive identification of miRNA coding sites on a whole-genome scale. Moreover, this offered vital insight into the function of miRNAs in replant disease. This study, based on the principal factors influencing replant disease formation (RDFs) identified in previous studies, established different treatments under controlled conditions, including NP, SP, FA, FO, and FAFO, and constructed corresponding sRNA libraries. The encoded site, precursor, and mature sequences of the *R. glutinosa* miRNA (*RgmiRNA*) were meticulously extracted from the *R. glutinosa* genome. A further analysis was conducted to investigate the cellular processes regulated by *RgmiRNAs*. On this basis, the expression profiling of *RgmiRNAs* was identified under different RDFs. From this, specific miRNAs associated with replant disease were identified. This study provides fundamental data for the in-depth analysis of the roles of miRNAs in *R. glutinosa* growth and development, stress response, and the biosynthesis of active components. Furthermore, it establishes a foundation for elucidating the function of non-coding RNAs in replant disease and proposing alleviated technology.

## 2. Materials and Methods

### 2.1. Plant Materials and Each Treatment

Two distinct soil types were extracted from the 25 cm layer of soil in two separate isolated pools: one that had never been planted with *R. glutinosa* and one that had only been planted with *R. glutinosa* once previously. These isolation plots were located at the Wen Agricultural Institute, Jiaozuo City, Henan Province, China. No specific permissions were required for this field experiment or the related activities. The methodology employed in the construction of the isolation plots has been previously described [4]. For the sake of convenience, the former soil was designated NP soil, and the latter was designated SP soil. Subsequently, both soils were transferred into plastic pots with a diameter of 25 cm and a height of 22 cm. A total of five treatments were conducted in pot experiments, comprising NP soils, SP soils, NP soils irrigated by FA, NP soils irrigated by FO [identified as *Fusarium oxysporum* f. sp. *R. glutinosa*] [12], and NP soils irrigated by a mixed application of FA with FO. Each treatment was assessed in 20 pots. The pot experiments were conducted in a controlled environment (28 °C, 10,000 lx, photoperiod of 14 h light/10 h dark) at the Institute of GAP for Chinese Medicinal Materials, Fujian Agriculture and Forestry University, China. 

*R. glutinosa* cultivars ‘Wen 85-5’, which exhibited the characteristic symptoms of replant disease, were used as the experimental materials. The *R. glutinosa* seedlings were initially cultivated in nutrient-rich soils using their tuberous roots as the propagating material. In summary, the tuberous roots of *R. glutinosa* were divided into several sections, each containing one or two bud eyes. The tuberous root-derived sections were planted in a nutrient-rich medium under controlled conditions (28 °C, 10,000 lx, photoperiod of 14 h light/10 h dark) until the development of four-leaf seedlings. Subsequently, the seedlings were transferred from the nutrient soil to the plastic pots containing different soil types. In accordance with the RDFs employed in previous studies [12], along with the quantity of soil present in the plastic pots and the characteristics of the soil, each treatment was conducted as follows. The pots designated for the FA treatment were irrigated using 1 mmol L^−1^ FA solution until a negative appearance, analogous to that induced by replant disease, was observed. The final concentration of FA in the soil was maintained at a maximum of 10 mmol L^−1^. The FO treatment was conducted using *F. oxysporum*, which was initially cultivated in 100 mL of potato dextrose broth (PDB) for a period of 4–5 days at 24 °C. Thereafter, the mycelia were extracted from the PDB solution cultivated *F. oxysporum* using four layers of gauze, and the resulting solution was diluted to 1 × 10^6^ conidia/mL with sterilized water. The FO suspension solution was applied to *R. glutinosa* until the disease symptoms became apparent. In the case of FAFO treatments, the aforementioned FA and FO solutions were applied simultaneously to *R. glutinosa* until the disease symptoms became apparent. The NP and SP treatments were irrigated with an identical volume of sterilized water. At 10, 20, 30, 40, and 50 days following each treatment, the roots of the different treatments were sampled, rapidly frozen in liquid nitrogen and stored at −80 °C for subsequent analysis. The samples after treatment exhibiting clear symptoms of replant disease were used to construct sRNA library.

### 2.2. Construction and Sequencing of the R. glutinosa sRNA Library

The total RNA was isolated from root samples subjected to the various treatments using TRIZOL methods. The quality and purity of the total RNA from each sample were evaluated using a NanoDrop 2000 spectrophotometer. The 18–35 nt small RNA (sRNA) was separated from the total RNA through 15% denaturing polyacrylamide gel electrophoresis (PAGE). The isolated small RNAs were subsequently ligated to the 5′ and 3′ ends of oligonucleotide adapters. The linked products were then reverse-transcribed with specific primers into cDNA, which was subsequently amplified by PCR. cDNA production was further screened on 15% denaturing PAGE, followed by sequencing on the Illumina HiSeq™ 2000 platform. In order to obtain clean reads of sRNA from the different samples, the raw reads from the sequencing platform were filtered to remove low-quality sequences, adapters, and polyN sequences. This was accomplished through the utilization of the Cutadapt v3.2 software [21].

### 2.3. Annotation of Clean Reads of R. glutinosa sRNA 

The sRNA clean reads were collapsed to obtain unique reads through the col-lapse_reads_md.pl scripts of the miRdeep2 v0.1.3 software [22]. The genome of *R. glutinosa* (NCBI Assembly accession number: ASM1608111v2 [20]) was obtained from the NCBI database. The clean reads were subsequently aligned to the *R. glutinosa* genome to identify sRNA reads derived from the *R. glutinosa* genome using the Bowtie 1.0 software (https://github.com/BenLangmead/bowtie, accessed on 20 May 2022) with no mismatches. In order to ascertain the category of small RNA (sRNA) to which the clean reads in different samples belong, the various sRNA datasets were downloaded from public databases. The snRNA and snoRNA sequences were obtained from the RNAcentral database (https://rnacentral.org/, accessed on 15 October 2023), the tRNA sequences from the GtRNAdb 2.0 database (http://lowelab.ucsc.edu/GtRNAdb/, accessed on 15 May 2022), and the repetitive DNA sequence from the Repbase database (V20.05, https://www.girinst.org/repbase/, accessed on 26 May 2022). The clean reads were then aligned in sequence to the aforementioned datasets, comprising rRNA, sRNA, snoRNA, tRNA, and Repbase, with the objective of verifying the types of sRNA. This was conducted using the Bowtie 1.0 software, with one mismatch permitted. Following the alignment of the clean reads in the various sRNA datasets and subsequent filtering, the remaining sequences were employed in the identification of miRNAs within the *R. glutinosa* genome.

### 2.4. Identification of miRNA Coding Sites in the R. glutinosa Genome

The sRNAminer v1.1.2 software [23] was used to identify potential miRNA family in the *R. glutinosa* genome, employing 30 sRNA libraries with the default parameters. The identified candidate *R. glutinosa* miRNAs, including their respective precursors, mature sequences, and star sequences (miRNA*), were collated. The quantifier.pl script, as presented in mirdeep2 [22], was then employed to ascertain the abundance of miRNA and miRNA* in the 30 samples. To ensure the reliability of the identified *R. glutinosa* miRNAs, the candidate miRNAs were validated following their expression levels across different treatments. Only those miRNAs or miRNAs* with a read count exceeding 100 in at least three samples were retained. Subsequently, these miRNAs were subjected to manual checking for validating the final *R. glutinosa* miRNAs. The DESeq2 v1.45.3 software was used to identify significantly differentially expressed miRNAs between SP or RDFs and NP that satisfied the following criteria: log2 (fold change)> or <−1, *p*-value < 0.05. The heatmap for miRNA expression and differentially expressed miRNAs was generated using the MeV 4.9 software [24].

### 2.5. Target Identification of R. gluitnosa miRNAs

The mRNA of selected samples including FA and FAFO was captured from total RNA through the use of beads attached to oligo(dT). The enriched mRNA fragments were then ligated with a 5′ RNA adapter. Subsequently, the linked products were subjected to reverse transcription using biotinylated random primers, followed by amplification through the use of PCR methods. The PCR products were subjected to further purification to obtain degradome libraries, which were then sequenced on an Illumina HiSeq 2500. The raw data obtained from sequencing was filtered to remove reads with adapters, low-quality sequences and uncertain base sequences, thus obtaining clean reads. Additionally, two degradome libraries [15] in a previous study were selected to identif the target genes of *R. glutinosa* miRNAs. The transcriptome constructed in the preceding study [4], which was revised according to *R. glutinosa* genome information, was employed to identify the target genes of the miRNAs. The target transcripts of the miRNAs were identified using CleaveLand 4.0 software [25] based on the four degradome libraries and the *R. glutinosa* transcriptome. Target sites with a *p*-value of less than 0.05 were considered reliable cleavage sites caused by the miRNA. The function of the target genes was subsequently investigated through the utilization of Nr annotation and GO annotation information.

### 2.6. Verification of the Relationship between Key miRNAs and Candidate Target Genes

The dual-luciferase reporter system is employed to substantiate the cleavage functions of pivotal *R. glutinosa* miRNAs in response to replant disease against their respective targets. Two vectors, pGreen-3′UTR-sensor and pGreen-GUS-competitor [26], were selected for the expression of the miRNA precursor and the sites of cleavage by miRNA in vivo, respectively. The precursor of the key miRNAs was cloned from the roots of *R. glutinosa* using primers with *Xho* I and *Eco*R I (Table S1 in the Supplementary File), and then inserted into the pEASY vector (Beijing TransGen Biotech Co., Ltd., Beijing, China) to form the pEASY-miRNA-Pre vector. The precursor sequences of the miRNAs were validated through Sanger sequencing. The pGreen-competitor plasmid was linearized using the restriction enzymes *Xho* I and *Eco*R I. The miRNA-predigested pEASY-miRNA-Pre was ligated into the pGreen-competitor vector, thereby forming the pGreen-competitor-RgmiRNA-Pre construct. The binding regions of the miRNAs on their respective targets were identified. Subsequently, the *Age* I and *Avr* II sites were incorporated into both ends of the binding regions, which were then biosynthesized in vitro. The biosynthesized fragments were subsequently introduced into the pGreen-3′UTR-sensor vector, which had been previously digested with *Age* I and *Avr* II. This process yielded the pGreen-3′UTR-RgTarget-sensor construct. A 20-nucleotide sequence of random sequence was introduced into the sites between *Age* I and *Avr* II in the pGreen-dualluc-Space-sensor vector as a negative control.

The pGreen-RgmiRNA-Pre-competitor, pGreen-3′UTR-RgTarget-sensor, and pGreen-Space-sensor vectors were introduced into the *Agrobacterium tumefaciens* (*A. tumefaciens*) GV3101 (pSoup) strain, and cultivated in LB plates with kanamycin (Kan). The positive clones were fully proliferated in liquid LB medium and subsequently collected by centrifugation at 4000 rpm for five minutes. The pellets of *A. tumefaciens* were resuspended in a mixture composed of 10 mM MES, pH 5.6, 10 mM MgCl₂, and 200 µM acetosyringone, respectively. The *A. tumefaciens* with pGreen-RgmiRNA-Pre were immediately mixed with the *A. tumefaciens* with pGreen-3′UTR-RgTarget-sensor, thereby forming the experimental group. The pGreen-RgmiRNA-Pre were then mixed with the pGreen-Space-sensor, thereby forming the control group. The prepared mixture was cultivated at 28 °C in the dark for a period of three hours. The experiment was conducted on *Nicotiana benthamiana* (*N. benthamiana*) with four leaves to ascertain the cleavage activity of the miRNAs on their target sites. In practice, the abaxial surfaces of the leaves were infiltrated using a 1-mL needle-free syringe with the *A. tumefaciens* mixture suspension. Each *N. benthamiana* plant with two leaves infected by *A. tumefaciens* was considered a single biological replicate, with three biological replicates for both the treatment and control groups. Following a 72 h cultivation period of the infected plant, the infiltrated leaves were harvested and processed using the Dual Luciferase Reporter Assay Kit DL101-01 (Vazyme Biotech Co., Ltd., Nanjing, China). Luciferase (LUC) and Renilla (REN) activities were quantified using a Promega GloMax 20/20 luminometer (Promega Biotech Co., Ltd., Fitchburg, WI, USA). The ratio of luciferase (LUC) to Renilla (REN) activity is employed as a means of characterizing the inhibitory effect of miRNAs on their respective targets.

### 2.7. The Expression Pattern Analysis of Key microRNAs and Their Target Genes

The step-loop method was employed for the quantification of miRNAs, and the step-loop primers were designed through CE Design v1.04 (Vazyme Biotech Co., Ltd., Nanjing, China). The total RNA from the various samples was employed as a template for reverse transcription using the miRNA 1st cDNA Synthesis Kit (Vazyme Biotech Co., Ltd., Nanjing, China). The 20 μL qPCR mixtures were prepared with 1 μL cDNA, 0.4 μL miRNA-specific primer (10 μM) (Table S1 in the Supplementary File), 0.4 μL mQ Primer R, 10 μL SYBR mixture (Vazyme Biotech Co., Ltd., Nanjing, China), and 8.2 μL deionized water. The following parameters were employed for the qPCR programme: the reaction was initially heated to 95 °C for 30 s, followed by 40 cycles of 95 °C for 10 s, and 59°C for 10 s. The abundance of miRNAs was normalized using *R. glutinosa* 18S as the reference gene. The relative expression of miRNAs was calculated using the 2^−ΔΔCT^ method [27], with *R. glutinosa* seedlings, before their transplantation into designated soils, which served as the control. The value of the control was set to 1.

The qPCR primers for target genes of *R. glutinosa* miRNA were manually designed. First-strand cDNA was synthesized from the total RNA by employing the HiScript II 1st Strand cDNA Synthesis Kit (Vazyme Biotech Co., Ltd., Nanjing, China). qPCR was then performed using a total reaction mixture of 20 μL, comprising 1 μL cDNA, 0.4 μL each of a pair of primers (10 μM) (Table S1 in the Supplementary File), 10 μL SYBR mixture (Vazyme Biotech Co., Ltd., Nanjing, China), and 8.2 μL deionized water. The qPCR programme was conducted in accordance with the aforementioned miRNA qPCR. Furthermore, the 18S gene of *R. glutinosa* was used to normalize the expression levels of the target genes across various samples. Subsequently, the relative expression of the target genes was calculated using the same methodology employed for miRNA expression analysis.

## 3. Results

### 3.1. Construction of sRNA Libraries of R. glutinosa in Relation to RDFs

**Figure 1:**
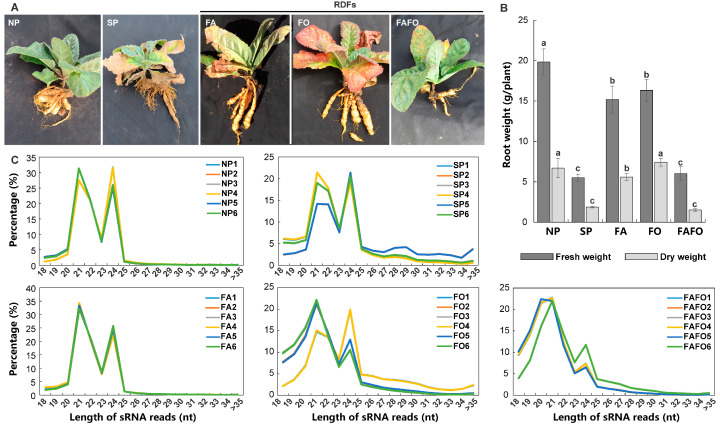
Construction of sRNA libraries of *R. glutinosa* treated with NP, SP, and RDFs (FA, FO, and FAFO). (**A**,**B**) Phenotypic characteristics and biomass changes of *R. glutinosa* roots under different treatments. (**C**) The length distribution of clean reads from sRNA libraries under different treatments and their replicates. The use of lower-case letters indicates that the observed differences are statistically significant (*p <* 0.05; LSD). The data are presented as the mean ± standard deviation (*n* = 3).

To accurately identify the specific miRNAs that respond to replant disease and to minimize the interference of miRNAs elicited by non-replant disease-related environmental factors, five treatments were established under controlled environments. The experimental design comprised three RDFs (FA, FO, and FAFO), one SP, and NP. The NP soils was employed as a control for the other treatments. The results demonstrated that the diverse treatments exerted a significant influence on the growth and development of *R. glutinosa*, when compared with NP (Figure 1A). At 30 days after transplantation (DATs), distinctive features were identified in *R. glutinosa* for the various treatments (Figure 1A). Nevertheless, the severity of the observed symptoms in the *R. glutinosa* plants varied depending on the specific treatment employed. The root formation of the SP *R. glutinosa* was most severely affected, exhibiting the most prominent fibrous roots (Figure 1A). The symptoms of FAFO are similar to those of SP; however, there are still notable differences between the two. The primary effect of FAFO is the elongation of tuberous roots, with relatively minimal damage to the aboveground portions. Furthermore, FA and FO demonstrated a diminished effect on root growth, while exhibiting pronounced damage to the leaves (Figure 1A). A subsequent analysis of the changes in root biomass of *R. glutinosa* under different treatments revealed that in comparison with the NP, the biomass of *R. glutinosa* treated with SP and FAFO was significantly reduced, while FA and FO had relatively minor effects on the root biomass (Figure 1B). *R. glutinosa* samples at 40 DATs were collected for the establishment of sRNA libraries. The number of raw reads generated in six replicates of each sample is presented in the following table: the number of raw reads ranged from 7.74 to 12.76 million in the NP, 8.91 to 13.35 million in the SP, 7.96 to 13.59 million in the FA, 9.82 to 17.53 million in the FO, and 8.58 to 13.61 million in the FAFO (Table S2 in the Supplementary File). Following quality control, the number of clean reads obtained from each sample ranged from 6.65 to 11.21 million for NP, 6.72 to 10.42 million for SP, 6.37 to 11.11 million for FA, 6.59 to 9.95 million for FO, and 6.14 to 10.14 million for FAFO (Table S2 in Supplementary File).

The length of the sRNA present in the aforementioned 30 sRNA libraries was determined. The results demonstrated that the clean reads in the various sRNA libraries exhibited lengths spanning from 21 to 25 nt (Figure 1C). The most prevalent sRNAs were those with lengths of 21 nt and 24 nt (Figure 1C). This distribution pattern is largely in accordance with the typical range of plant sRNA lengths (Figure 1C). In order to obtain clean reads originating from the *R. glutinosa* genome, the clean reads of all samples were initially aligned to the *R. glutinosa* genome. The results demonstrated that there were 4.79–7.65 million clean reads in NP, 3.13–4.61 million in SP, 4.47–7.84 million in FA, 1.98–3.53 million in FO, and 1.42–2.89 million in FAFO that were mapped to the *R. glutinosa* genome (Table S3 in Supplementary File). The mapped clean reads were subsequently annotated using non-coding sRNA datasets, including rRNA, snRNA, snoRNA, and tRNA, extracted from the public database. The proportion of clean reads that were successfully annotated ranged from 11.23% to 18.24% in NP, 12.83% to 17.18% in SP, 9.73% to 27.18% in FA, 10.58% to 17.39% in FO, and 7.12% to 18.52% in FOFA (Table S3 in Supplementary File). In order to mitigate the influence of these reads on the identification of miRNAs, they were eliminated. The remaining clean reads were employed for the identification of the miRNA family in *R. glutinosa*.

### 3.2. Identification of the miRNA Family Existing in the Genome of R. glutinosa

**Figure 2:**
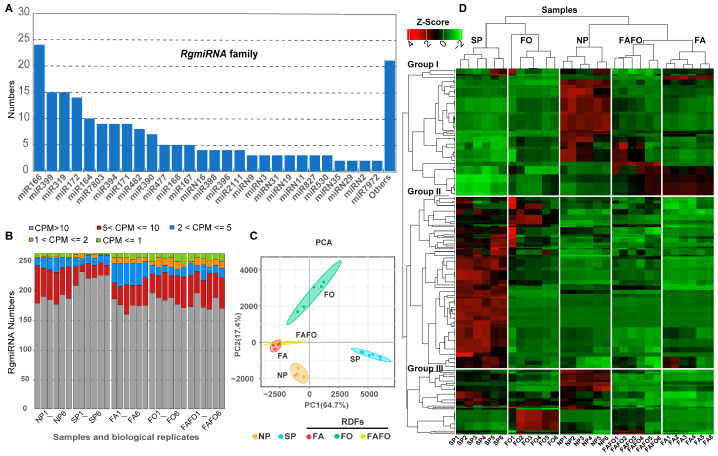
*RgmiRNAs* identified in the genome of *R. glutinosa* and their expression profiling under NP, SP, and RDFs. (**A**) *RgmiRNA* family and their members within the genome of *R. glutinosa*. (**B**) The overall distribution of the abundance of *RgmiRNA* in different samples. (**C**) PCA analysis of *RgmiRNA* abundance derived from different sRNA libraries. (**D**) The expression levels of *RgmiRNAs* in the various samples. The expression levels of all miRNAs in the heatmap were transformed using the Z-Score method.

In order to comprehensively identify the miRNAs of *R. glutinosa*, 30 sRNA libraries were utilized to explore the miRNA sites within the *R. glutinosa* genome by means of the sRNA-miner v1.1.2 software [23]. As a result, 343 miRNA loci were identified within the *R. glutinosa* genome. Subsequently, the expression abundance of all miRNAs was calculated using mirdeep2 [22]. To obtain a more accurate representation of the *R. glutinosa* miRNAs, only those miRNAs or miRNA* that had mapped clean reads of more than 100 in at least three samples out of thirty samples were retained as candidate *R. glutinosa* miRNAs. Subsequently, 201 miRNAs were identified as eligible for classification as *R. glutinosa* miRNAs (*RgmiRNAs*) (Table S4 in the Supplementary File). Of these, 160 were identified as conserved miRNAs, while 41 were not identified in other plants and are therefore regarded as novel or specific miRNAs in *R. glutinosa* (Table S4 in the Supplementary File). These *RgmiRNAs* were classified into 49 distinct miRNA families, as detailed in Table S4 in the Supplementary File. The *RgmiR166*, *RgmiR399*, *RgmiR319*, and *RgmiR172* family was the most abundant, comprising more than 14 members (Figure 2A and Table S4 in the Supplementary File). Subsequently, 10, 9, 9, 9, 8, and 7 members were identified, respectively, for the *RgmiR164*, *RgmiR7803*, *RgmiR394*, *RgmiR171*, *RgmiR482*, and *RgmiR390* families (Figure 2A and Table S4 in Supplementary File). It is noteworthy that the *RgmiR477*, *RgmiR168*, *RgmiR167* family comprises five members (Figure 2A and Table S4 in Supplementary File). The remaining 36 miRNA families comprised between one and four individual members (Figure 2A and Table S4 in the Supplementary File).

In order to gain a comprehensive understanding of the expression levels and patterns of all *RgmiRNAs*, the abundance of each mature *RgmiRNA* and its star (RgmiRNA*) sequence was calculated based on the number of clean reads per million mapped reads (CPM) (Table S5 in Supplementary File). A comprehensive analysis of *RgmiRNA* expression across diverse samples demonstrates that the expression of *RgmiRNAs* is relatively uniform among biological replicates within a single sample. However, conspicuous differences in expression patterns emerge between samples (Figure 2B; Table S5 in Supplementary File). A principal component analysis (PCA) of the *RgmiRNA* abundance from the various samples revealed that SP was distinctly separated from the other samples. FAFO and FA demonstrated crossover and aggregation (Figure 2C). To demonstrate the expression profile of *RgmiRNAs* across different samples, a heatmap was constructed using the expression matrix of all *RgmiRNAs*, with Z-score normalization and hierarchical clustering (Figure 2D). The clustering of the five samples indicates that the SP samples were conspicuously grouped into a single category, while the FA, FO, and FAFO samples were grouped into another category (Figure 2D). This also implies that the effects of SP and RDFs on the expression of miRNAs are markedly dissimilar. Furthermore, SP is considerably more complex, encompassing all of the replant disease-related stress factors and their interactions. 

The expression levels of *RgmiRNAs* in different samples were employed to classify the *RgmiRNAs* expression pattern into five groups (Figure 2D). Group I comprise 91 *RgmiRNAs*, some of which exhibited higher expression levels in NP, FA, and FAFO than in the other samples (Figure 2D). Group II comprises 125 *RgmiRNAs*, majority of which exhibited higher expression levels in SP in comparison with the other groups (Figure 2D). Furthermore, some *RgmiRNAs* displayed elevated expression levels in FO relative to other groups. It is noteworthy that the abundance of this group of *RgmiRNAs* in SP was significantly higher than in other treatments, indicating that these miRNAs may be specifically expressed in SP samples (Figure 2D). Furthermore, group III contains 47 *RgmiRNAs*, some of which were predominantly expressed in SP, FO, and NP in comparison with other treatments (Figure 2D). Furthermore, 72 RgmiRNAs* were identified as having higher abundance in various samples (Table S5 in the Supplementary File), indicating that both mature miRNAs and their star sequences with higher abundance may also exert a significant role in *R. glutinosa*. The miRNA star sequence will be the focus of further analysis in subsequent studies.

### 3.3. Identification and Functional Analysis of R. glutinosa RgmiRNAs Target Genes 

**Figure 3:**
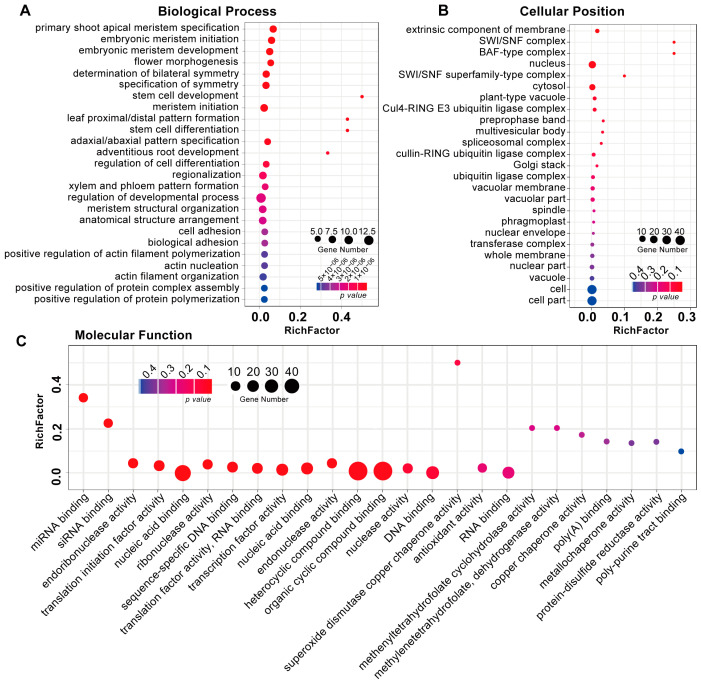
Analysis of GO categories encompassing biological process (**A**), cellular position (**B**), and molecular function (**C**) for candidate target genes of *RgmiRNAs*.

In order to investigate the function of *RgmiRNAs* in *R. glutinosa*, NP and FAFO samples were selected for the establishment of a degradome library. The results revealed that 16.20 million and 11.58 million raw reads were obtained from the two samples, respectively. Following quality filtering, 15.55 million and 10.18 million clean reads were obtained from each sample. Furthermore, two degradome libraries from a previous study [15] were selected to identify the targets of *RgmiRNAs*. Concurrently, a transcriptome established in a previous study was further revised following the genome information of *R. glutinosa*, to identify targets of *RgmiRNA*. The candidate targets of *RgmiRNAs* were primarily obtained through Cleaveland 4.0 [25], based on the four degradomes and the transcriptome of *R. glutinosa*. Consequently, 107 *RgmiRNAs* or RgmiRNAs* were identified to target 189 transcripts (Table S6 in the Supplementary File). To gain further insight into the cellular pathways associated with *RgmiRNAs*, the function of the transcripts targeted by *RgmiRNAs* was investigated through gene ontology (GO) analysis. The GO enrichment analysis revealed that the 189 transcripts were involved in three functional groups (Figure 3). The predominant biological processes were found to be the ‘regulation of developmental process’, ‘xylem and phloem pattern formation’, ‘meristem structure organisation’, and another category (Figure 3A). In the category of cellular position, the most significantly enriched groups were ‘cell part’, ‘nucleus’, ‘cytosol’, and others (Figure 3B). With regard to molecular function, the most highly enriched groups were ‘organic cyclic compound binding’, ‘heterocyclic compound binding’, ‘nucleic acid binding’, and others (Figure 3C). These findings indicate that miRNAs are involved in the regulation of diverse cellular pathways and exert pivotal functions during the growth and development of *R. glutinosa*.

### 3.4. Analysis of Differentially Expressed miRNAs in RDFs

**Figure 4:**
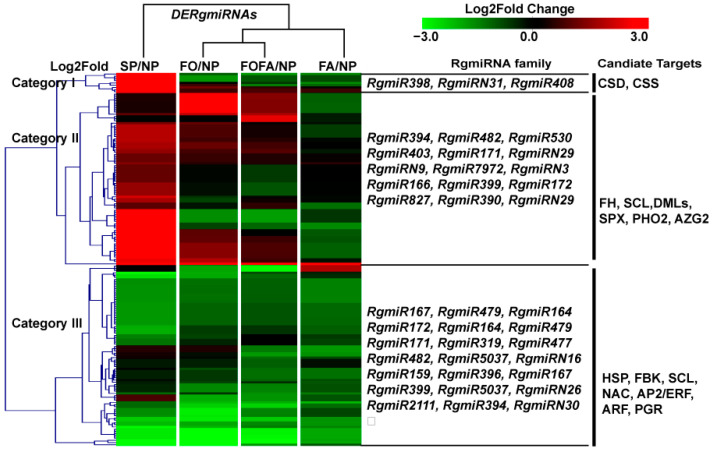
Identification of differentially expressed *RgmiRNAs* (*DeRgmiRNAs*) in *R. glutinosa* treated with SP and RDFs in comparison with those treated with NP. In the identification of *DeRgmiRNAs*, NP was employed as the control, while SP and RDFs were subjected to treatment. The heat map provides a visual representation of the distribution of *DeRgmiRNAs* across the various treatments. The *DERgmiRNA* family and their target genes identified in the degradome are displayed on the right side of the heat map.

In order to gain insight into the role of *RgmiRNAs* in the formation of replant disease, differentially expressed *RgmiRNAs* (*DeRgmiRNAs*) were identified in various SP and RDFs in comparison with NP. A total of 124 *DeRgmiRNAs* were identified in SP, with 70 exhibiting increased expression and 54 displaying decreased expression (Table S7 in the Supplementary File). In FA, a total of 86 *DeRgmiRNAs* were identified, comprising 4 upregulated and 82 downregulated (Table S8 in the Supplementary File). Similarly, FO exhibited 86 *DeRgmiRNAs*, comprising 25 upregulated and 61 downregulated sequences (Table S9 in the Supplementary File). Finally, a total of 90 *DeRgmiRNAs* were identified in FAFO, comprising 14 upregulated and 76 downregulated (Table S10 in the Supplementary File). A heatmap and cluster analysis were conducted to illustrate the distribution of *DeRgmiRNAs* among different samples (Figure 4). The results demonstrated that the clustering pattern of *DeRgmiRNAs* across samples was essentially identical to that of *RgmiRNAs*.

It is worthy of note that *RgmiRNAs* in category 1 exhibited overexpression exclusively in SP, as exemplified by *RgmiR398c*, *RgmiR398b*, *RgmiR398d*, and *RgmiR408*, with negligible or no expression in the other treatments (Figure 4). In particular, the expression of *RgmiR398c* exceeded 3000 CPM in the SP samples, while it was less than 10 CPM in NP and RDFs (Figure 4, Table S5 and S7–S10 in the Supplementary File). This indicates that *RgmiR398* is abnormally concentrated in SP and is associated with the development of replant disease. These miRNAs have been observed to primarily target genes encoding copper/zinc-superoxide dismutase (CSD) and copper/zinc-superoxide dismutase copper chaperone precursor (CSS). The category 2 *RgmiRNAs* were found to be upregulated in SP, FO, and FAFO, including *RgmiR394*, *RgmiR171*, *RgmiR166*, *RgmiR399*, *RgmiR827*, *RgmiR7972*, and so forth. This category of *RgmiRNAs* was observed to target genes including scarecrow-like protein (SCL), SPX domain-containing membrane protein (SPX), transcriptional activator DEMETER (DMLs), and so forth (Figure 4). The miRNAs in category 3 exhibited a notable decline in expression levels in SP and different RDFs. This group primarily comprises *RgmiR172*, *RgmiR2111*, *RgmiR164*, *RgmiR319*, and so forth. These miRNAs have been observed to target NAC transcription factors (NAC), AP2/ERF transcription factors (AP2/ERF), and F-box/kelch-repeat proteins (FBK) (Figure 4). In general, category 1 comprises a relatively limited number of *DERgmiRNAs*. However, *RgmiR398*, which is specifically expressed in SP, mainly targets genes related to the oxidation balance. It can thus be postulated that *RgmiR398* may be intimately associated with the regulation of the oxidation balance of replant disease of *R. glutinosa*.

### 3.5. Expression Changes of RgmiR398/RgCSD in Replant Disease and RDFs

**Figure 5:**
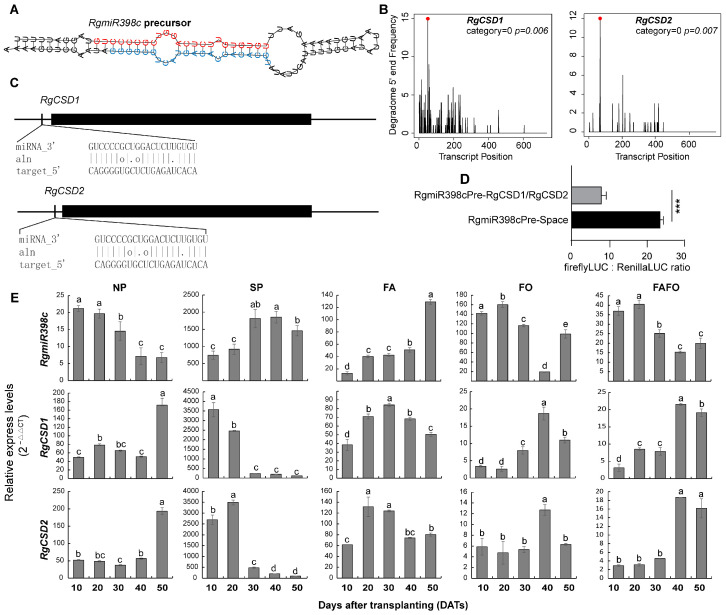
Verification of *RgmiR398* and its target genes, as well as analysis of their expression characteristics during the treatment of various stress. (**A**) Structural representation of the *RgmiR398c* precursor. (**B**) The cleavage of *RgmiR398c* against *RgCSD* was identified through degradome analysis. (**C**) The cleavage sites of *RgmiR398* on *RgCSD* were illustrated in a diagrammatic representation. (**D**) The target site of *RgmiR398* in *RgCSD* was further validated through a dual luciferase reporter gene assay. (**E**) The expression pattern of *RgmiR398c* and the target *RgCSD* during different treatments. *R. glutinosa* seedlings prior to transplantation into the experimental pots for treatment were employed as controls, and the expression level was set at 1, which was not depicted in the figure. The use of lower-case letters indicates that the observed differences are statistically significant (*p* < 0.05; LSD). *** indicates a significant difference at the 0.001 level of confidence. The data are presented as the mean ± standard deviation (*n* = 3).

To elucidate the regulatory impact of *RgmiR398* on the oxidative balance of replant *R. glutinosa*, the *RgmiR398c* precursor was cloned following the predicted *RgmiR398c* precursor (Figure 5A). The results demonstrated that the *RgmiR398c* precursor cloned in *R. glutinosa* exhibited near-perfect identity with the theoretical prediction, with only two mismatch bases. The *RgmiR398c* precursor exhibits all the properties expected of an miRNA precursor. Concurrently, *RgmiR398c* was found target *R. glutinosa CSD* genes (*RgCSD*) in the degradome (Figure 5B). The two *RgCSD* genes were successfully cloned from *R. glutinosa*. The lengths of the two *RgCSDs*, designated *RgCSD1* and *RgCSD2*, was 880 and 767 base pairs (bp), respectively, and both exhibited complete coding regions (Figure 5C). To validate the targeting relationship between *RgmiR398c* and *RgCSD*, a dual luciferase assay was employed to assess the cleavage ability of *RgmiR398c* on *RgCSD*. The results demonstrated that *RgmiR398c* was capable of effective cleavage within the 5′ UTR regions of *RgCSD1* and *RgCSD2* (Figure 5D). These findings indicate that *RgmiR398* plays a role in regulating the oxidation system of replanted *R. glutinosa*.

The expression characteristics of *RgmiR398c* and two *RgCSDs* were subjected to further in-depth analysis under NP, SP, and RDFs. The findings revealed that the *RgmiR398c* and *RgCSD* genes exhibited distinguishable expression profiles in response to the diverse treatments. *RgmiR398c* in *R. glutinosa* plants grown in NP soils displayed high expression levels at 10 DATs, followed by a gradual decline. While the targeted *RgSOD1* and *RgSOD1* demonstrated relatively lower expression at 10–40 days after transplanting (DATs), as illustrated in Figure 5E. The expression of *RgmiR398c* in *R. glutinosa* treated with SP soils demonstrated a gradual increase at 10 DATs, whereas its two targeted *RgCSDs* exhibited an inverse expression trend. In the context of FA stress, *RgmiR398c* exhibited lower expression levels at 10–40 DATs, followed by a rapid increase. In contrast, the two target genes exhibited higher expression levels at 10–40 DATs (Figure 5E), indicating that allelochemical autotoxicity had a limited impact on *RgmiR398c* during the initial stages of *R. glutinosa* development. In response to FO stress, *RgmiR398c* demonstrated a notable elevation in expression after 10–20 DATs, which subsequently declined. The targeted *RgCSD* displayed an inverse trend (Figure 5E). Furthermore, the expression of *RgmiR398c* displayed a comparable trend to that of the FO treatment under FAFO conditions. *RgmiR398c* displayed higher expression levels at the initial stages of the treatment, followed by a decline at subsequent stages (Figure 5E). This suggests that FO and FAFO treatments significantly induce the expression of *RgmiR398c* at an earlier stage.

## 4. Discussion

In response to a multitude of stress factors, plants meticulously regulate the expression of a series of miRNAs. These particular response miRNAs are present in multiple forms and undergo significant alterations in expression levels, thereby functioning as pivotal regulators of plants’ responses to a wide variety of stress factors [28,29,30]. The development of replant disease is a complex process that involves a range of principal factors, including allelochemicals, microorganisms, and their interactions. Consequently, replant disease exhibits both common biotic and abiotic stress characteristics, while also displaying distinctive properties. It is therefore of the utmost importance to elucidate the formation mechanism of replant disease through a detailed interpretation of the common and specific miRNAs in response to replant disease. This study constructed 30 sRNA libraries associated with SP and RDFs in a controlled environment. In conclusion, a total of 201 miRNA loci were accurately identified within the *R. glutinosa* genome. Moreover, a number of crucial *DeRgmiRNAs* were identified in SP and RDFs when compared with NP. The expression pattern analysis of *RgmiRNAs* and *DeRgmiRNAs* revealed that the impact of replant soils on *R. glutinosa* miRNAs was markedly distinct from that of single RDFs. The analysis of *RgmiRNA* target genes indicated that replant soils and RDFs exerted a specific influence on the normal growth and development of *R. glutinosa*. Moreover, replant disease led to a significant disturbance in the ROS equilibrium in *R. glutinosa*, which was mediated by *RgmiR398*. The results of the miRNA analysis under replant disease and RDFs provided crucial data that further elucidated the formation mechanism of replant disease in *R. glutinosa*.

In response to various types of stress, plants have been observed to modulate the expression of specific miRNAs [30]. In this study, a notable decline in the expression of *RgmiRNAs* was observed in response to SP and RDFs. This indicates that SP and RDFs may impede essential cellular processes in *R. glutinosa* by inhibiting miRNAs. Among the *RgmiRNAs* that were inhibited by SP and RDFs, miR172 plays a pivotal role in regulating the size of plant roots, stems, leaves, and fruit, as well as the development of floral organs [31,32,33]. The overexpression of Arabidopsis miR172 has been demonstrated to result in a significant increase in root length [34]. In potato, the overexpression of *miR172* has been observed to facilitate tuber expansion [35]. This suggests that there is a positive correlation between miR172 expression and the formation of plant organs. miR2111b is closely associated with the processes of root morphology and structure [36,37]. The overexpression of legume *miR2111b* has been demonstrated to result in a significant increase in root density and root diameter [38]. The miR164-NAC regulatory module is involved in the development of roots, the uptake of nutrients, and the adaptation to stress. In Arabidopsis and maize, miR164 reduces the sensitivity of auxin and inhibits lateral root development by degrading *NAC* [39]. The upregulation of wheat *miR164* has been demonstrated to suppress *NAC1*, which subsequently inhibits the formation of new adventitious roots [40]. The downregulation of *RgmiR164* in a variety of treatments indicates that SP and RDFs promote the development of adventitious roots in *R. glutinosa*. These findings suggest that SP and RDFs may potentially inhibit the root development of replanted *R. glutinosa* through the reduction of *RgmiR2111b*, *RgmiR172*, and *RgmiR164* levels. The preceding studies have demonstrated that both replant disease and RDFs exert an influence on the root growth of *R. glutinosa* [12,41]. Furthermore, an increase in fibrous roots is a primary consequence of replant disease [4]. It is hypothesized that these repressed miRNAs may be closely related to the manifestation of *R. glutinosa* symptoms in response to diverse stress treatments.

Specific stress factors not only inhibit the expression of specific miRNAs but also enrich key miRNAs. It was observed that SP and RDFs significantly promoted the accumulation of specific *RgmiRNAs*. It is noteworthy that the number of significantly upregulated miRNAs in SP was found to be higher than in single RDFs. *miR827* has been demonstrated to target two SPX-containing region proteins (SPX), thereby confirming its role in the absorption and storage of phosphorus (P) [42]. The findings of this study demonstrate that *RgmiR827* is markedly upregulated in SP. *R. glutinosa*. This indicates that the SP soil disordered the absorption and metabolism of nutrients in *R. glutinosa* via *RgmiR827*. Furthermore, another upregulated *miR399* in replant *R. glutinosa* was identified as a regulator of P metabolism, exerting its influence on the expression of the *UBC* gene [43,44]. The overexpression of *miR399* has been observed to result in root P poisoning [43]. The present study demonstrated that *RgmiR399* exhibited a specific upregulation in response to SP, whereas it displayed a downregulation or no significant change in other RDFs. This suggests that replant disease may have a significant impact on the nutritional metabolism of *R. glutinosa* through specific miRNAs. miR7972 could indirectly determine the regulation of gene expression by targeting DEMETER-like DNA glycosylase (DML) [45]. DML, a DNA damage repair enzyme, plays a pivotal role in the dynamic regulation of DNA methylation levels [46,47]. The expression of *RgmiR7972* in replant *R. glutinosa* indicates its involvement in the regulation of gene expression in replant *R. glutinosa*. miR171 primarily targets *SCL* genes and is extensively implicated in diverse cellular processes, including the maintenance of the shoot apical meristem, the transition between growth phases, and stress response [48,49]. The overexpression of Arabidopsis *miR171* has been observed to result in a reduction in branch number, root length, and leaf shape [50]. The expression of *RgmiR171* was observed to be repressed in *R. glutinosa* replant, indicating that replant disease may affect the development of *R. glutinosa* tissues and organs by upregulating specific miRNAs.

It is noteworthy that *RgmiR398* was significantly upregulated in *R. glutinosa* plants subjected to replant disease, while no notable changes were observed in RDFs. Moreover, the expression of *RgmiR398* in *R. glutinosa* subjected to replant disease exhibited aberrant accumulation in comparison with other RDFs. *RgmiR398* was identified as a target of two *RgCSDs*, suggesting that replant disease may potentially disrupt the expression of core members of the antioxidant enzyme system through *RgmiR398*. As a result, this has an impact on the functionality of the antioxidant enzyme system of *R. glutinosa*. A study of the changes in ROS and antioxidant enzyme activities during the formation of replant disease in *R. glutinosa* revealed a gradual increase in ROS content and a corresponding decrease in superoxide dismutase (SOD) activity [12,51]. The expression changes of *RgmiR398* and *RgCSD* in response to RDFs were analysed. It was demonstrated that RDFs could also exert a significant influence on the alteration of *RgmiR398*. The FA treatment was conducted in the later stage, while the FA and FAFO treatments were conducted in the early stage. Concurrently, the impact of FA, FO, and FAFO on *RgmiR398* was markedly inferior to that of replant disease. This is consistent with previous studies, indicating that different RDFs significantly impact the oxidation balance of *R. glutinosa*, although the extent of this alteration varies [12]. These findings indicate that SP exerts a more pronounced effect on the oxidative damage of *R. glutinosa* than RDFs. Previous studies on replant *R. glutinosa* have demonstrated a correlation between oxidative balance and the development of replant disease [4,12]. Several studies on various plants with replant disease characteristics, including *Solanum melongena*, *Lilium davidii, Angelica sinensis*, *Codonopsis tangshen,* have provided evidence to support this conclusion [52,53,54,55]. It has been demonstrated that oxidative imbalance plays a significant role in the decline of plants subjected to replant disease. The precise mechanism by which oxidative imbalance occurs in replant disease remains uncertain. The present study has identified *RgmiR398*, which is specifically upregulated in *R. glutinosa* subjected to replant disease, as a regulator of antioxidant enzyme activity. This finding establishes a crucial point of entry for elucidating the mechanisms of replant disease-induced oxidative imbalance. It provides a foundation for further insights into the death processes in replant plants.

Moreover, this study demonstrated that a considerable number of *RgmiRNAs* displayed a significant upregulation in replant disease and a notable downregulation in RDFs. However, the target genes of these *RgmiRNAs* remained unidentified within the degradome. These *RgmiRNAs* may play a pivotal role in replant disease, and further investigation is required to elucidate their precise functions. A comparison of *RgmiRNAs* expression in response to different treatments indicates that SP soils and single RDFs both influence *RgmiRNAs* expression. However, notable discrepancies in the expression patterns across treatments have been observed. Although the combined treatment of FA and FO (FAFO) involved two RDFs, this is still quite different from actual replant disease, which involves a large number of RDFs interacting with one another. Furthermore, the impact of these RDFs on replant disease is not merely additive; rather, there is a sophisticated interplay between them. Consequently, replant disease represents a highly distinctive stressor, differing from general stressors. A specific set of response *RgmiRNAs* is thus activated during the process of replant disease formation. 

## 5. Conclusions

In conclusion, in this study, the *RgmiRNA* family was first identified on a whole-genome scale, along with the determination of their candidate target genes. The identification of these miRNAs in *R. glutinosa* provides a valuable data resource for the in-depth clarification of the roles of *RgmiRNAs* during replant disease formation, growth and development, stress response, and the biosynthesis of active components in *R. glutinosa*. Furthermore, this study elucidates the expression profiles of *RgmiRNAs* under NP, SP, and RDFs, identifying specific *RgmiRNAs* in response to replant disease. Additionally, the potential roles of *RgmiR398/RgCSD* in the formation of oxidative stress of *R. glutinosa* subjected to replant disease were proposed. These findings significantly enhance our comprehension of the functions of miRNAs in intricate multi-factor stress.

## Data Availability

The original contributions presented in the study are included in the article and Supplementary Material, further inquiries can be directed to the corresponding author.

## Supplementary Materials

The following supporting information can be downloaded at: https://www.mdpi.com/article/10.3390/genes15091239/s1, Table S1. Primer lists utilized in this study; Table S2. Statistics of sRNA raw reads, filtered reads, and clean reads generated from different *R. glutinosa* sRNA libraries; Table S3. The annotation of clean reads from *R. glutinosa* sRNA libraries under different treatments in non-coding sRNA database; Table S4. *RgmiRNAs* identified in the genome of *R. glutinosa*; Table S5. Expression abundance (CPM) of *RgmiRNAs* in *R. glutinosa* under different treatments; Table S6. Candidate transcripts targeted by *RgmiRNAs* identified in the degradome; Table S7. *DeRgmiRNAs* in SP compared with NP *R. glutinosa*; Table S8. *DeRgmiRNAs* in FA compared with NP *R. glutinosa*; Table S9. *DeRgmiRNAs* in FO compared with NP *R. glutinosa*; Table S10. *DeRgmiRNAs* in FAFO compared with NP *R. glutinosa*.
